# Sensitivity of imaging for multifocal-multicentric breast carcinoma

**DOI:** 10.1186/1471-2407-8-275

**Published:** 2008-09-30

**Authors:** Anna Bozzini, Giuseppe Renne, Lorenza Meneghetti, Giuseppe Bandi, Gabriela Santos, Anna Rita Vento, Simona Menna, Stefania Andrighetto, Giuseppe Viale, Enrico Cassano, Massimo Bellomi

**Affiliations:** 1 Breast Imaging Unit, Department of Radiology, European Institute of Oncology, Via Ripamonti 435 - 20141 Milan, Italy; 2 Department of Pathology and Laboratory Medicine, European Institute of Oncology, Via Ripamonti 435 - 20141 Milan, Italy; 3 Ospedale Civile degli Infermi di Vigevano, C.so Milano 5 - 27029 Vigevano, Italy; 4 Department of Senology, European Institute of Oncology, Via Ripamonti 435 - 20141 Milan, Italy; 5 Department of Radiology, European Institute of Oncology, Via Ripamonti 435 - 20141 Milan, Italy; 6 School of Medicine, University of Milan, Via Festa del Perdono 7 - 20122 Milan, Italy

## Abstract

**Background:**

This retrospective study aims to determine: 1) the sensitivity of preoperative mammography (Mx) and ultrasound (US), and re-reviewed Mx to detect multifocal multicentric breast carcinoma (MMBC), defined by pathology on surgical specimens, and 2) to analyze the characteristics of both detected and undetected foci on Mx and US.

**Methods:**

Three experienced breast radiologists re-reviewed, independently, digital mammography of 97 women with MMBC pathologically diagnosed on surgical specimens. The radiologists were informed of all neoplastic foci, and blinded to the original mammograms and US reports. With regards to Mx, they considered the breast density, number of foci, the Mx characteristics of the lesions and their BI-RADS classification. For US, they considered size of the lesions, BI-RADS classification and US pattern and lesion characteristics. According to the histological size, the lesions were classified as: index cancer, 2nd lesion, 3rd lesion, and 4th lesion. Any pathologically identified malignant foci not previously described in the original imaging reports, were defined as undetected or missed lesions. Sensitivity was calculated for Mx, US and re-reviewed Mx for detecting the presence of the index cancer as well as additional satellite lesions.

**Results:**

Pathological examination revealed 13 multifocal and 84 multicentric cancers with a total of 303 malignant foci (282 invasive and 21 non invasive). Original Mx and US reports had an overall sensitivity of 45.5% and 52.9%, respectively. Mx detected 83/97 index cancers with a sensitivity of 85.6%. The number of lesions *un*detected by original Mx was 165/303. The Mx pattern of breasts with undetected lesions were: fatty in 3 (1.8%); scattered fibroglandular density in 40 (24.3%), heterogeneously dense in 91 (55.1%) and dense in 31 (18.8%) cases. In breasts with an almost entirely fatty pattern, Mx sensitivity was 100%, while in fibroglandular or dense pattern it was reduced to 45.5%. Re-reviewed Mx detected only 3 additional lesions. The sensitivity of Mx was affected by the presence of dense breast tissue which obscured lesions or by an incorrect interpretation of suspicious findings.

US detected 73/80 index cancers (sensitivity of 91.2%), US missed 117 malignant foci with a mean tumor diameter of 6.5 mm; the sensitivity was 52.9%

Undetected lesions by US were those smallest in size and present in fatty breast or in the presence of microcalcifications without a visible mass.

US sensitivity was affected by the presence of fatty tissue or by the extent of calcification.

**Conclusion:**

Mx missed MMBC malignant foci more often in dense or fibroglandular breasts. US missed small lesions in mainly fatty breasts or when there were only microcalcifications. The combined sensitivity of both techniques to assess MMBC was 58%. We suggest larger studies on multimodality imaging.

## Background

Multifocal-multicentric tumors of the breast (MMBC) are defined by the presence of two or more physically separate neoplasms in the same breast. Pathologists define multiple, simultaneous primary lesions when there are two or more foci of tumors without intervening neoplastic tissue [1,2]. They are defined as multifocal when only one breast quadrant is involved and multicentric when two or more quadrants are involved [1,3]. An exact "radiological definition" does not exist, but tumors are usually considered as multifocal when the distance is less than or equal to 5 cm and multicentric when the distance is more than 5 cm between lesions [4]. Given the lack of anatomically distinct borders between the breast quadrants, and the difficulty in radiologically evaluating the actual distance between lesions, for the purpose of the current investigation multifocal and multicentric tumors were collectively identified as MMBC.

The estimated prevalence of MMBC is between 4 and 65% of all breast carcinomas; this variability is mainly due to lack of standardization in the gross examination and sampling of breast specimens [5,6]; furthermore, sampling may be limited to the areas with suspicious macroscopic findings, or may be extensively performed, sometimes using whole mount sections.

Several reports have shown that the sensitivity of mammography (Mx) and ultrasound (US) for detecting multiple malignant foci is about 50% [4,7-14], while the sensitivity of Magnetic Resonance Imaging (MRI) is higher in detecting MMBC, reaching 94–99% sensitivity for invasive ductal carcinoma (IDC) and 50–80% for ductal intraepithelial neoplasia (DIN) [10,15].

This retrospective study aims at assessing the sensitivity of preoperative Mx and US and of re-reviewed mammograms to detect MMBC identified at pathological examination of surgical specimens, as well as analyzing the characteristics of both detected and undetected foci on Mx and US and the sensitivity of both methods.

## Methods

From October 2000 to October 2004, 1129 (14.5%) of 7769 breast cancers treated at the European Institute of Oncology in Milan were diagnosed as MMBC.

Among these, we selected 97 patients who undertoke pre-operative digital imaging Mx and for whom the complete series of images were retrievable. Eighty of them (82%) were also examined by US. The median age of the patients with MMBC was 48 years (range 26–78 years). Sixteen (17%) patients were treated pre-operatively with neo-adjuvant chemotherapy and presurgical Mx and US were evaluated.

Fifty-six (58%) patients underwent mastectomy and 41 (42%) breast-conserving surgery. For macroscopic sampling we used the reference standard of serial 5-mm slicing of the surgical specimen (lumpectomy or mastectomy) following Egan's methods [16]. Pathological evaluation was performed on tumor blocks sampled from any suspicious areas. The histopathologic examination of the specimens had been performed independently by the 2 breast pathologists who are co-authors of this paper.

Tumors were defined as MMBC when at least 5 mm of normal tissue was interposed between the two lesions [17]. For all identified neoplastic lesions, the histological features, including size, tumor type, grade, presence of intraepithelial neoplasia (including lobular intraepithelial neoplasia [LIN] which has disagreeing pathologic interpretations especially in regard to multifocal lesions) were recorded and correlated with the original Mx report. The pathologically identified malignant foci not previously described in the original imaging report, were defined as undetected lesions. All lesions were recorded and ranked according to size: "index cancer" designated as the one seen at Mx, which prompted the operation, followed by 2nd lesion, 3rd lesion, and 4th lesion. The mean histologic tumor size was measured for the index cancer as well as for each additional lesion. The mean distance, taking into consideration, the longest and the shortest distance between neoplastic foci was also determined. Three expert radiologists, with more than 5 years experience in breast imaging, re-reviewed independently all the mammograms, after being informed of the histopathological findings. Mx had been performed with digital equipment (GE SENOGRAPHE 2000 D, Milwaukee, WI, USA). Routine mediolateral, oblique and craniocaudal mammograms were obtained in all patients, and whenever required by the radiologist, additional spot-compression magnification and true-lateral images were performed, to better study the suspicious findings and the extent of disease. Each breast was classified into one of the four following groups according to its density as defined by the BI-RADS lexicon 1) breast with the density of fat, 2) fatty breast with scattered fibroglandular densities, 3) heterogeneously dense breast, 4) dense breast [18-27]. We evaluated the number of lesions, their Mx features and classified the lesions with respect to: microcalcifications (elements, distribution, and number), mass lesions (shape, margin, and density), microcalcifications in a mass, architectural distortions and focal asymmetry of glandular tissue. The largest pathological diameter was used as the lesion size.

The microcalcifications were further classified according to the BI-RADS lexicon: benign or amorphous, scattered, diffuse, in multiple clusters (BI-RADS<= 3); amorphous, pleomorphic, heterogeneous, with distribution in clusters, segmental or regional (BI-RADS 4); allomorphic, heterogeneous, linear, branching with linear or segmental distribution (BI-RADS 5).

Based on the description of mass lesions, the lesions were classified according to BI-RADS: round, oval with circumscribed margins and equal or low density (BI-RADS 3); lobular, irregular with microlobulated, obscured, indistinct margins and equal or rather high density (BI-RADS 4); irregular with indistinct, spiculated margins and high density (BI-RADS 5). "Architectural distortion": the normal architecture is distorted with no definite mass visible (BI-RADS 4–5). Focal asymmetric breast tissues were classified as BI-RADS 4 [27,28].

Eighty out of 97 (82.5%) patients were also examined by US. US was performed using a 10–14-MHz linear-array probe (Esaote Technos, Genoa, IT). Suspicious areas were also scanned by radial and antiradial orientation with and without compression. US assessment was based on reports and the images were re-reviewed by the same three expert radiologists in breast imaging. All the US reports were appropriate to the corresponding images. In the US reports relating to images, the following features were considered: echo pattern, shape, boundary and margin of the lesions (Table 1). The index cancer and subsequent lesions were evaluated and descriptions classified according to BI-RADS: oval, isoechoic with circumscribed margins, sometimes having an echogenic halo, posterior acoustic enhancement or none (BI-RADS 3); irregular, hypoechoic with abrupt interface and without posterior acoustic shadowing (BI-RADS 4–5); the same sonographic assessment but with the presence of microcalcification in the lesion (BI-RADS 5). All lesions detected by Mx and US were classified according to BI-RADS lexicon: BI-RADS code 5: highly suggestive of malignancy; BI-RADS code 4: suspicious for malignancy; BI-RADS code 3: probably benign; BI-RADS code 2 benign; BI-RADS code 1 negative [18,27]. Lesions categorized as BI-RADS 1, 2 and 3 were considered missed in the sensitivity calculations.

**Table 1:** Breast density for US lesions.

	Φ	**Foci ** **pathology** **detected**	**Det ** **US**	**UD ** **US**	**Dense ** **Breast**	**Heterogeneous ** **Dense Breast**	**Fatty ** **breast**	**Fatty with ** **fibroglandular** **tissue...**
1	23,8 mm(2–70)	80	73	7	2	1	3	1
2	8,7 mm(0,1–39)	80	35	45	8	3	18	16
3	6,6 mm(0,1–20)	46	15	31	4	2	15	10
4	6,6 mm(0,1–39)	45	10	35	9	5	17	4

Sensitivity was calculated for Mx and Us to detect the index and additional cancers: true positive\true positive + false negative.

The imaging characteristics of the lesions were analyzed together with the specific sensitivity. A case report form (CRF), composed of three sections was prepared for recording data of the lesions. The first CRF section included the pathological findings, the second the data from the original imaging reports and the last section included the findings from mammogram re-review and the subsequent observations of the radiologist who re-reviewed the cases. In order to perform statistical analysis and calculate sensitivity, the data were also recorded in a customized digital data base (Access 97, Microsoft^®^, Redmond, WA, USA). Statistical analysis was carried out to determine whether the detection rate of MMBC at Mx and at US was related to the size of the index cancer or to breast density.

## Results

Pathological examination of the 97 histopathologic specimens revealed 303 neoplastic foci (282 invasive and 21 intra-epithelial). The mean number of foci per patient was 3. Neoplastic lesions were: invasive ductal carcinoma NOS in 215 (71%) cases, invasive lobular carcinoma in 57 (19%) cases, ductal intraepithelial neoplasia in 16 (5%) cases, lobular intraepithelial neoplasia (LIN2) in 5 (1.6%) cases, invasive micropapillary and tubular carcinoma in 10 (3.2%) cases (Table 2).

**Table 2:** Sensitivity of mammography and number of lesions detected and undetected at the original Mx report according to the histological type: IDC = Invasive ductal carcinoma; ILC = invasive lobular carcinoma; other IC = invasive carcinoma of special type; DIN = ductal intraepithelial neoplasia; LIN = lobular intra-epithelial neoplasia;

	**N.**	**Mx detected**	***Mx Undetected***	**Sensitivity (%)**
**IDC**	215	110	*105*	51.2
**ILC**	57	21	*36*	36.8
**other IC**	10	3	*7*	30
**DIN**	16	4	*12*	25
**LIN**	5	//	*5*	0.0

**Tot**	303	138	*165*	*45.5*

The mean tumor size was 25 mm for the index cancer (range: 2 – 77), 9 mm for the second lesion (range 8–39 mm), and 6.6 mm (range 1–25 mm) for the additional lesions (Table 3). The mean distance, between neoplastic foci was 10 mm, 80 mm and 5 mm (Table 4). Mx detected 83/97 index cancers (85.6% sensitivity), 38/97 2nd lesions (39.2% sensitivity), 11/55 3rd lesions (21.8% sensitivity), 3/33 4th lesions (12.1% sensitivity), 3/23 further lesions (13.0% sensitivity).

**Table 3:** Number of undetected lesions at the original Mx report according to breast pattern and lesion rank (1° = index cancer)

**Lesion rank**	**mean diameter and range**	***Total number of UD Mx lesions***	**Dense Breast (%)**	**Heterogeneous Dense Breast **(%)	**Fatty Breast with scattered Fibroglandular densities (%)**	**Breast with the density of fat (%)**
index lesion	25 mm (2–77)	*14*	3 (21.4)	8 (57.2)	3 (21.4)	0 (0.0)
2^nd^	9 mm (0.1–39)	*61*	12 (19.7)	31 (50.8)	17 (27.9)	1 (1.6)
3^rd^	6.9 mm (0.1–20)	*44*	6 (13.6)	25 (56.8)	12 (27.3)	1 (2.3)
4^th^	6.8 mm (0.1–39)	*29*	6 (20.7)	17 (58.6)	5 (17.2)	1 (3.5)
≥ 5^th^	6.3 mm (0.1–25)	*17*	4 (23.5)	10 (58.8)	3 (17.7)	0 (0.0)

Total		*165*	31 (18.8)	91 (55.1)	40 (24.3)	3 (1.8)

*(UD Mx = Lesions undetected at mammography)*.

**Table 4:** Range of the distance between each of the lesions and all of the other satellite lesions measured at histopathology.

**Range of Histological distance Intra lesion (mm)**	**Index lesion**	**2**^nd^	**3**^rd^	**≥ 4**^th^
**Range**	**5 – 80**	**5 – 80**	**5 – 25**	**5**

For index cancers, the evaluation of Mx BI-RADS was 14 (15%) BI-RADS 1; 10 (10%) BI-RADS 3; 30 (31%) BI-RADS 4; 43 (45%) BI-RADS 5. Mx missed 165 malignant foci with a mean size of 7.6 mm. Overall sensitivity for Mx was 45.5% (138/303) (Table 5).

**Table 5:** Number of lesions detected by Mx at the original report and at re-review of mammograms with their BI-RADS (Mx) class according to re-review rank.

**Lesion ** **rank**	**Number of ** **lesions** **detected by** **Pathology**	**Number of ** **lesions ** **detected by** **original MX**	**Mx BIRADS**					**Number of ** **lesion** **detected at** **re-revision**	**Sensitivity ** **%**	**Mean ** **diameter at** **Pathology **
						
			**1**	**2**	**3**	**4**	**5**			
Index lesion	97	83	1	0	1	3	4	**84**	85.6	25
2nd	97	38	4	1	0	0	3	38	39.2	9
3rd	55	11	5	0	5	1	1	**12**	20.0	6.9
4th	33	3	9	0	1	5	7	**5**	9.1	6.8
>5th	23	3	4	-	0	6	4	3	13.0	6.3
6th			4		-	2	1			
7th			3			1	2			
8th			0							
9th			2							
			0							

Tot	303	138						**141**	45.5	//

The patterns of lesions defined at Mx re-review were as follows: 32 microcalcifications, 51 mass lesions, 46 microcalcifications in masses, 8 distortions and 4 glandular asymmetries (Table 6). The histological type of carcinomas undetected by Mx was invasive ductal carcinoma NOS in 105 (63.6%) cases, invasive lobular carcinoma in 36 (21.8%) cases, ductal intraepithelial neoplasia in 12 (7.2%) cases, lobular intraepithelial neoplasm in 5 (3.03%) cases, and invasive micropapillary and tubular carcinoma in the 7 (4.2%) remaining cases (Table 2).

**Table 6:** Characteristics of the lesions seen at re-review of the mammograms according to their rank.

**Lesions**	**Index lesion**	**2**^nd^	**3**^rd^	**4**^th^	**5**^th^	**ALL**
Microcalcifications	18	9	3	1	1	32
Mass feature	30	16	2	2	1	51
Microcalcifications+Mass	28	15	3	-	-	46
Distortion	5	2	1	-	-	8
Other	3	1	-	-	-	4

total	84	43(38)	9(12)	3(5)	2(3)	141

The breast pattern of those undetected Mx lesions was: fatty breast in 3 (1.8%) cases, fatty breast with scattered density in 40 (24.3%) cases, heterogeneous dense breast in 91 (55.1%) cases, and dense breast in 31 (18.8%) cases (Table 3).

In our series, Mx sensitivity of breasts with an almost entirely fatty pattern was 100%, while in fibroglandular or dense pattern, sensitivity is reduced to 46% (Table 7).

**Table 7:** Pattern and sensitivity of mammography in the index lesion related to their BI-RADS class.

	**BIRADS**								
	
	**ALL**	**1**	%*	**3**	%*	**4**	%*	**5**	%*
Entirely fatt	2	-	-	-	-	1	1.0	1	1.0
Scattered fibroglandular densities	26	4	4.1	3	3.1	10	10.3	9	9.3
Heterogeneously dense	55	9	9.3	4	4.1	16	16.5	26	26.8
Extremely dense	14	4	4.1	1	1.0	3	3.1	6	6.3

	97	17		8		30		42	

In breast with an almost entirely fatty pattern, the sensitivity of Mx was 100% while in dense breast sensitivity was 40.5%.

Re-review of mammograms detected 3 additional lesions not reported at the original examination: one index cancer and two 4th lesions (Table 5). These lesions were defined as masses with margins obscured by superimposing adjacent tissue which was isodense with fine amorphous calcifications (BI-RADS 4). The 3 additional lesions were in heterogeneous dense breasts.

US detected abnormal imaging as solid lesions with ill defined or irregular borders, non calcified lesions with posterior acoustic shadowing, solid intracistic or intraductal lesions.

We based a patient's final imaging diagnosis on the combination of mammographic and sonographic findings.

US detected 73/80 index cancers (91.2% sensitivity), 35/80 2nd lesions (43.8% sensitivity), 16/46 3rd lesions (34.8% sensitivity), 10/45 further lesions (22.2% sensitivity). For index cancers, the evaluation of US BI-RADS were 9 (11%) BI-RADS 1; 2 (3 %) BI-RADS 3; 28 (35) BI-RADS 4; 41 (51%) BI-RADS 5 [27]. US missed 117 malignant foci with a mean tumor diameter of 6.5 mm. The sensitivity was 52.9% (133/251) (Table 8).

**Table 8:** Sensitivity of US and number of lesions detected by US according to lesion rank and size with their BI-RADS code and US pattern: hypo-echoic (hypo); iso-echoic (iso)

**Lesion rank**	**Mean histologic diameter**	**Total number of lesions detected by pathologists**	**Number of lesions detected by US**	**BIRADS US**					**Hypo**	**Iso**	**Sensitivity (%)**
							
				**1**	**2**	**3**	**4**	**5**			
Index lesion	25	80	73	7	-	4	28	41	71	2	91.2
2nd	9	80	35	45	-	3	16	16	33	2	43.8
3rd	6.9	46	15	31	-	1	7	7	15	-	32.6
≥ 4th	6.5	45	10	35	-	2	4	4	8	2	22.2

Tot	//	251	133								52.9

US missed the smallest lesions that were close in proximity to the largest lesion in fatty breast or when there were microcalcifications without a US visible lesion mass (Table 4, 9). Clusters of microcalcifications or some linear or segmental microcalcifications are not visible at US for the lack of a lesion mass.

**Table 9:** Size (mean, *median *and range in millimeters) and rank of lesions undetected by US, Mx and re-reviewed mammograms

Lesion rank	Undetected by US	Undetected by Mx	Undetected by re-Rev Mx
Index lesion	23.8	15.8	14.7
	***15***	***11.5***	***11***
	2 – 70	3 – 52	3 – 52

2^nd^	8.8	8.4	8.4
	***6***	***6***	***6.4***
	0.1 – 39	0.1 – 39	0.1 – 39

3^rd^	6.6	6.6	6.1
	***5***	***5***	***4.5***
	0.1 – 20	0.1 – 20	0.1 – 20

4^th^	6.2	6.8	6.8
	***4***	***4***	***4***
	0.2 – 39	0.1 – 39	0.1 – 39

≥ 5^th^	6.3	5.7	5.7
	***3***	***2.5***	***2.5***
	0.1 – 25	0.1 – 25	0.1 – 25

The re-review gave the same results only based on the reports corresponding to the US images.

## Discussion

The accurate definition of multifocal and multicentric breast carcinoma according to the distribution of the neoplastic foci within the same or different quadrants of the affected breast is hampered by the lack of anatomically defined borders between the quadrants and it gives no precise information about the intervening distance. The different definitions may be responsible for the wide range of prevalence of MMBC reported in literature [6]. The current TNM classification criteria (AJCC/UICC), refers to diameter of the largest lesion mass for the T classification of multifocal-multicentric carcinomas, thus underestimating the total tumor volume and understaging these tumors [14,19]. However, if the sum of the diameters of all tumor masses is taken into account, then the metastatic pattern of MMBC does not significantly differ from that of unifocal tumors of the same size of the sum [21,22]. Moreover, Brenin and Morrow demonstrated a higher incidence of axillary lymph node metastases in MMBC than unifocal tumors of the same pT class [20]. For these reasons we think that any neoplastic foci should be measured to verify Fish's statement: "mean size of unifocal tumors was smaller than multifocal cases when an aggregate measurement of foci was utilized" [24].

Accurate pre-operative information about the occurrence, size and location of multifocal lesions is a prerequisite for a proper breast conserving surgical management.

Mx has been established as the fundamental breast imaging modality.

In our study the most frequent Mx pattern was characterized by heterogeneous dense breast (encountered in 56.7% of the patients), while a fatty pattern was observed in only 2.1%. In the literature Mx is most sensitive for detection of index cancer in fatty breasts, while the sensitivity decreases in breasts with fibroglandular or dense patterns.

In our series, sensitivity of Mx was 85.6% in the detection of the index cancer, but reduced to 45.5% in the detection of all the neoplastic foci. It is also important to emphasize that the re-review of mammograms did not significantly increase the overall sensitivity in detecting MMBC, confirming that, while the sensitivity for identifying the index cancer is high, it decreases for the smaller additional foci. This indicates that in dense breasts the smallest foci cannot be distinguished [25].

The re-review of mammograms detected 3 additional lesions: one index cancer and two 4th lesions, described as masses with margins obscured by adjacent tissue that was isodense with fine amorphous calcifications.

Following prior definitions in the literature, lesion patterns were defined at Mx re-review as: 32 microcalcifications, 46 microcalcifications in nodules, 51 masses, 8 distortions and 4 others, confirming that even small clusters of microcalcifications are well detected in dense breasts. The mean diameter of the pathology rank was 2 and 6.3 mm, for the index cancer and ultimate satellite lesion, respectively (Table 5). The mean size of the lesions undetected by Mx re-review is 11 and 2.5 mm, for the index cancer and ultimate satellite lesion, respectively (Table 9).

The most common causes of undetected breast cancer by Mx include: above all, dense parenchyma obscuring masses particularly in the absence of microcalcifications followed by technical errors and incorrect interpretation of suspicious findings [29,30]. These results are in line with the conclusions of Mendelson et al., who evaluated breast density as a predictor of mammographic lesion detection [31].

US is a useful complementary method in detecting additional tumors that escaped Mx detection [13,14]. US may detect small masses in glandular tissue but is not as helpful in fatty tissue or in the characterization of morphology or the extent of calcifications [29,31].

In our series dealing with a majority of dense breasts, the global sensitivity of US is 52.9%, being 91.2% for the index lesion. The use of US in addition to Mx, increases global sensitivity to 58% in assessing multifocality (Table 10).

**Table 10:** Sensitivity of US, Mx and combined sensitivity of Mx + US

	**Foci pathology detected**	**Foci detected by US**	**Foci detected by Mx**	**Foci detected by Mx and/or US**	**Sensitivity US %**	**Sensitivity MX %**	**Sensitivity MX + US %**
**Index Lesion**	80	73	69	77	91	86	96
**2**^nd^	80	35	31	43	44	39	54
**3**^rd^	46	15	9	15	33	20	33
**> 4**^th^	45	10	6	10	22	13	22

**Σ**	251	133	115	145	52	46	58

According to the literature, mammographic-sonographic sensitivity does not exceed 60–63% [30], thus we can then speculate that undetected malignant foci in the remaining breast tissue after breast-conserving surgery should not affect the overall survival rates of the patients, as indicated by the randomized trials comparing mastectomy with conservative surgery [32]. Post-operative radiotherapy has a major role in reducing the risk of ipsilateral tumor recurrence in conservatively treated patients [33]. These arguments could question the need for an accurate pre-operative diagnosis of MMBC using MRI to detect all satellite foci around the main lesion to exactly determine the impact of imaging on the recurrence rate and on the overall survival of patients with breast cancer: ad hoc clinical trials should be designed to answer this question.

## Conclusion

Mx and US missed 165 and 118 of 303 malignant foci, respectively. Mx missed small malignant foci of MMBC especially in dense or fibroglandular breasts. Re-review of the Mx detected only a few additional lesions and did not significantly increase the sensitivity of the examination. US missed small lesions, close to the largest lesion, in fatty breasts. US also missed cancers presenting as Mx microcalcifications without a mass. The combined sensitivity of both techniques was 58%.

The need for an accurate pre-operative assessment of patients with MMBC is currently debated, due to the uncertain effect of multifocality or multicentricity on the recurrence rate and on the overall survival of patients treated with breast-conserving surgery and post-operative radiotherapy.

In summary, whole breast sonography is the first-line complementary imaging modality to mammography in the preoperative examination of women. Considering that the global sensitivity for assessing multifocality is 58%, we suggest more and larger studies on multimodality imaging (US and Mx and then MRI in comparison) and long term observation periods to determine the influence on the incidence of recurrence and on the overall prognosis of patients with breast cancer.

## Abbreviations

AJCC: American Joint Committee on Cancer; BIRADS: Breast Imaging Reporting and Data System; CRF: Case Report Form; DIN: Ductal Intraepithelial Neoplasia; IC: Invasive Carcinoma; IDC: Invasive Ductal Carcinoma; ILC: Invasive Lobular Carcinoma; LIN: Lobular Intraepithelial Neoplasia; MMBC: Multifocal-Multicentric Breast Carcinoma; MRI: Magnetic Resonance Imaging; MX: Mammography; NOS: Not Otherwise Specified; TNM classification: primary Tumor, regional lymph Nodes and distant Metastases classification system; UICC: Union Internationale Contre le Cancer/International Union Against Cancer; US: Ultrasound.

## Competing interests

The authors declare that they have no competing interests.

## Authors' contributions

AB: devised the study, participated in the design, carried out the re-reviewed mammograms and US and drafted the manuscript. GR: participated in the conception and design of the study, carried out the histopathologic examinations and helped in drafting the manuscript. LM and GB: carried out the re-reviewed mammograms and US and helped drafting the manuscript. GS and ARV: carried out surgical data and helped in drafting the manuscript. SM and SA : collected the statistical data, performed the statistical analysis and helped draft the manuscript. GV: carried out the histopathologic examinations and helped draft the manuscript. EC and MB: participated in the conception and the design of the study and critically reviewed the manuscript. All authors read and approved the final manuscript.

## Ethics

Our Institute is a Comprehensive Cancer Center classified by the Italian Ministry of Heath as ¿IRCCS¿ (Institute for Research and Care with Scientific Purposes), this means we are at the same time a Research institute and an Hospital. Each patient who refers to our institute for diagnosis, tratment or care, upon his/hers first visit is asked to sign a generic consent form. This consent form informs the patient that the anonimised clinical data of their exams and procedures might be used for scientific (i.e. clinical studies, retrospective studies and so on) and/or educational purposes (the Institute hosts many Postgraduate Schools of the University of Milan).

## Pre-publication history

The pre-publication history for this paper can be accessed here:


